# Nitrogen addition accelerated straw *in-situ* decomposition by promoting specific microbial taxa growth and straw decomposing enzyme activities

**DOI:** 10.3389/fpls.2025.1703916

**Published:** 2025-12-17

**Authors:** Tengfei Guo, Mengyuan Wang, Yulu Chen, Ke Yue, Long Ma, Shaomin Huang, Xinpeng Xu, Xiao Song, Sumiao Su, Zekun Zhang, Qian Zhang, Keke Zhang

**Affiliations:** 1 Institution of Plant Nutrition and Environmental Resources, Henan Academy of Agricultural Sciences, Zhengzhou, China; 2 State Key Laboratory of Efficient Utilization of Arid and Semi-arid Arable Land in Northern China, Ministry of Agriculture and Rural Affairs, Institute of Agricultural Resources and Regional Planning, Chinese Academy of Agricultural Sciences, Beijing, China; 3 Key Laboratory of Plant Nutrition and Fertilizer, Ministry of Agriculture and Rural Affairs, Institute of Agricultural Resources and Regional Planning, Chinese Academy of Agricultural Sciences, Beijing, China; 4 Resources and Environment College, Henan Agricultural University, Zhengzhou, China; 5 Institution of Edible Fungi, Henan Academy of Agricultural Sciences, Zhengzhou, China

**Keywords:** biomarker taxa, microbial community, N fertilization, straw decomposition, straw properties

## Abstract

**Introduction:**

Crop residue represents the largest input of organic carbon in agricultural ecosystems and its decomposition is fundamentally mediated by soil microbial communities. However, the mechanism of N fertilization regulating decomposition of the plant residue especially the associated key microbial taxa remain unclear.

**Methods:**

To address this gap, we conducted a 100-day field decomposition experiment using the litterbag method to track temporal shifts in straw physicochemical properties and associated microbial communities under three N regimes: no nitrogen (N0), 200 kg N ha^-1^ (N200), and 300 kg N ha^-1^ (N300).

**Results and discussions:**

Results showed that nitrogen addition significantly accelerated the decomposition of wheat straw, increasing mass loss and the degradation rates of cellulose, hemicellulose, and lignin relative to N0 treatment. Enzyme activities linked to carbon acquisition, including α-glucosidase (AG), β-glucosidase (BG), cellobiohyrolase (CBH), and β-xylosidase (XYL), were consistently elevated under N-amended treatments during mid- to late-stage decomposition. Similarly, activities of N-acquiring enzymes (β-N-acetyl-glucosaminidase, NAG; leucine aminopeptidase, LAP) and oxidative enzymes (polyphenol oxidase, PPO; laccase) were significantly enhanced, particularly after Day 14. Microbial community succession was tightly coupled with decomposition progression. Random forest modeling identified key bacterial biomarkers (e.g., *Terribacillus*, *Bacillus*, *Solibacillus*, *Oceanobacillus*, and *Cellulosimicrobium*) and fungal biomarkers (e.g., *Neocosmospora*, *Actinomucor*, *Fusarium*, *Chaetomium*, and *Aspergillus*), all of which are known for their capacity to degrade lignocellulosic and recalcitrant substances. Variation partitioning revealed that straw properties, especially the C/N ratio, TN content, and CBH activity, collectively explained the majority of microbial community variation. These findings support a mechanistic pathway in which nitrogen fertilization reduces residue C/N, thereby reshaping microbial community composition and stimulating enzyme production, which in turn accelerates decomposition. Our study provides novel insights into how nitrogen management influences the coupling of microbial ecology and biogeochemical cycling during straw decomposition, with direct implications for optimizing N fertilization management and sustaining soil fertility in agroecosystems.

## 1 Introduction

Globally, approximately 2.5 billion tons of crop straw are produced each year (FAO, 2017). Crop residues are widely recognized as a primary and essential source of organic matter in agricultural soils, particularly within the framework of modern farming systems (Bisen and Rahangdale, 2017). In agroecosystems, substantial volumes of straw are generated as byproducts of cereal crops such as wheat, contributing to the maintenance of soil organic matter and the provision of nutrients for subsequent cropping circles (Wang et al., 2020). Incorporating these residues into soil has proven to be an effective and environmentally sustainable strategy for improving soil’s physical, chemical, and biological properties, thereby supporting long-term food security (Haas et al., 2022). The decomposition of crop residues following their return to soil involves a complex series of microbial-driven processes that govern the mineralization and transformation of organic compounds (Paterson et al., 2008; Marschner et al., 2011). Evidence indicates that bacteria and fungi account for roughly 90% of total organic matter decomposition in such systems (Wahdan et al., 2023). Given the central role of microbial communities in mediating straw decomposition, sustained research efforts have focused on characterizing the temporal dynamics of these communities during decomposition and identifying the environmental and biochemical factors that regulate their activity.

A substantial body of research has examined how plant residue decomposition progresses alongside shifts in microbial community structure (Wegner and Liesack, 2016; Zhao et al., 2019). For instance, the biochemical breakdown of plant litter follows a sequential pattern: In the initial phase, readily degradable fractions such as water-soluble substances and labile polysaccharides including hemicellulose and non-shielded cellulose undergo swift breakdown; in contrast, structurally resilient materials like lignin and suberin remain largely intact during this period, undergoing mineralization only in subsequent stages (Voříšková and Baldrian, 2013). Nutrient availability and chemical composition of the substrate strongly influence the trajectory of microbial community assembly (Bastian et al., 2009). As decomposition advances, microbial taxa and their functional roles shift in response to evolving substrate quality. It has been reported that gram-negative (G-) bacteria mainly target labile C pools, whereas gram-positive (G+) bacteria and fungi exhibit a greater capacity to metabolize complex polymers such as cellulose and lignin (Li et al., 2020). Fungi, in particular, can modify lignin by oxidizing aromatic side chains, thereby contributing to the accumulation of more recalcitrant carbon forms. Gao et al. (2016) further confirmed that microbial succession during residue decomposition is closely linked to distinct biochemical phases, each marked by characteristic changes in enzymatic activity and community composition. Therefore, the structure of microbial assemblages during decomposition is shaped not only by temporal progression but also by the chemical nature of the organic C present in the residue. Despite these advances, a more comprehensive and mechanistic understanding of microbial community dynamics on decomposing straw remains necessary. The decomposition of straw by microbial consortia is influenced by multiple factors, including nutrient availability and the biochemical quality of the litter (Zhou et al., 2016). As the necessary management practice for crop productivity, N fertilization application also significantly affected the residue decomposition process by shaping soil microbial communities (Vivanco and Austin, 2011; Zhong et al., 2015). However, this influence is not uniform; it varies depending on the fertilization dose and soil native microbial community structure (Hobbie et al., 2012; Ma et al., 2024).

Straw exhibits a high carbon/nitrogen (C/N) ratio, which can constrain decomposition rates and potentially reduce yields in subsequent crops because of microbial immobilization of soil N (Eagle et al., 2000). Previous studies indicate that the inherently high C/N ratio of lignocellulosic residues elevates C availability in amended soils, promoting microbes to accelerate the mineralization of native soil organic matter to acquire N and maintain cellular stoichiometric balance (Chen et al., 2014; Liu et al., 2023). This phenomenon, known as microbial N mining, may intensify competition for available N between crops and decomposer communities, potentially limiting plant growth (Zhang et al., 2025). Adequate N fertilizer input can alleviate “microbial N limitation”, enhance the activity of hydrolytic enzymes targeting polysaccharides, and promote the rapid utilization of labile C. These shifts decrease the C/N ratio of decomposing residues and accelerate the breakdown of high-quality organic matter by enzyme activities (Bonanomi et al., 2019). Song et al. (2018) highlighted N addition promoting litter decomposition by enhancing invertase, β-glucosidase and polyphenol oxidase activity. Large N fertilization input were reported to slow residue decomposition by reducing decomposer abundance and suppressing the activities of oxidative enzyme for C, N-cycling enzymes (Entwistle et al., 2018). Recent studies increasingly emphasize the functional importance of specific key decomposers in regulating decomposition trajectories (Zheng et al., 2021). Nevertheless, critical knowledge gaps persist regarding how microbial communities, including taxonomic biomarkers, respond to varying N fertilization regimes and regulate enzymatic processes during straw decomposition under field conditions with different N fertilization.

To address these issues, we conducted a field experiment using the litterbag method to monitor wheat straw decomposition under three N fertilization rates. We hypothesize that nitrogen fertilization input influence straw-decomposition microbial communities and enzyme activities, which in turn accelerates the straw decomposition. In order to test this hypothesis, we assess temporal dynamics in straw mass loss and chemical composition; characterize the successional patterns of bacterial and fungal communities and identify taxonomic biomarkers associated with decomposition stages; and elucidate the mechanistic role of N fertilization in regulating decomposition dynamics.

## 2 Materials and methods

### 2.1 Experimental site

The study was conducted in 2023 at an experimental field located in Yuanyang County (34°47′45″N, 113°40′18″E), Henan Province, China. This region is characterized by a wheat-maize rotation system, which represents the dominant cropping pattern in the local agricultural landscape. The region experiences a continental monsoon climate, ranging from temperate to warm temperate, with a mean yearly temperature of 14.5°C and average annual rainfall totaling 615.1 mm. Soils in the area originate from Yellow River alluvium and are taxonomically designated as Calcaric Cambisols under the FAO classification framework. The initial soil had a pH of 8.35, 9.90 g kg^-1^ SOC, 1.22 g kg^-1^ total N, 20.57 mg kg^-1^ available phosphorus and 176.94 mg kg^-1^ available potassium.

### 2.2 Experimental design and sampling

The experiment was conducted using microplots (1.2 m×1.2 m), spatially distributed in a randomized layout within the field. Maize (10 plants per microplot) was sown on 6 June 2023 and harvested on 26 September 2023. Three nitrogen fertilization treatments were applied: no nitrogen fertilizer (N0), 200 kg N ha^-1^ (N200), and 300 kg N ha^-1^ (N300), which respectively represented the insufficient N, proper N and overdose N input in local agricultural production practice. All plots received a uniform basal application of 100 kg P_2_O_5_ ha^-1^, 90 kg K_2_O ha^-1^, and 7, 500 kg/ha of wheat straw. The tested wheat straw had 42.58% total C, 0.53% total N, 26.46% cellulose, 29.55% hemicellulose and 18.73% lignin, respectively.

Straw decomposition was monitored using the litterbag method. Fresh wheat straw was cut into 3–5 cm segments, oven-dried at 65°C for 24 h, and weighed before placing into nylon mesh bags (15 cm × 10 cm, 47 μm mesh size). Such small mesh size would prevent soil particles from mixing with the straw residues while allowing access by soil microorganisms, which necessarily means that our study focused on microbially mediated decomposition (Wang et al., 2012; Zhang et al., 2025). Litterbags were vertically inserted into the soil at a depth of approximately 15 cm. Three replicate bags per treatment were retrieved at 3, 7, 14, 30, 60 and 100 days after maize planting. Upon collection, bags were carefully cleaned of adhering soil and transported to the laboratory. Each residue sample was partitioned into three subsamples for distinct analytical purposes. One subsample was oven-dried at 65°C to constant weight to calculate mass loss, C and N content, and concentrations of cellulose, hemicellulose, and lignin. A second subsample was stored at 4°C for subsequent analysis of enzyme activities, DOC, and DTN. The third subsample was preserved at −80°C for DNA extraction and high-throughput sequencing of microbial communities.

### 2.3 Measurements of the straw properties

Carbon and nitrogen levels in straw samples were quantified via an elemental analyzer (Elementar Analysensysteme GmbH, Hanau, Germany). Cellulose, hemicellulose, and lignin contents were determined according to a revised detergent-based fiber protocol, adapted from the original Van Soest and Wine (1967) technique and further refined as detailed in Gao et al. (2016).

To quantify dissolved organic carbon (DOC) and dissolved total nitrogen (DTN), 0.20 g of dried residue was suspended in 30 mL of deionized water, then agitated in darkness at 25°C for 24 h. The supernatant was subsequently quantified using a TOC analyzer.

The catalytic activity of hydrolytic enzymes, specifically α-glucosidase (AG, EC 3.2.1.20), β-glucosidase (BG, EC 3.2.1.21), N-acetyl-glucosamine (NAG, EC 3.2.1.30), β-Cellobiosidase (CBH, EC 3.2.1.91), β-xylosidase (XYL, EC 3.2.1.37), and leucine aminopeptidase (LAP, EC 3.4.11.1) was evaluated using fluorogenic substrates based on 7-amino-4-methyl-coumarin (AMC) and 4-methyl-umbelliferone, following the protocol described by DeForest (2009). Fluorescence signals were detected by a multimode microplate reader, with excitation at 365 nm and emission at 450 nm. Activities of oxidation enzymes, namely polyphenol oxidase (PPO) and laccase, were measured using commercially available assay kits (Grace Biotechnology, Suzhou, China). For laccase, activity was determined by tracking the oxidation of 2, 2’-azino-bis (3-ethylbenzothiazoline-6-sulfonic acid) (ABTS) in a citrate-phosphate buffer system (100 mM citrate, 200 mM phosphate, pH of 5.0), with absorbance monitored at 420 nm (Zheng et al., 2021).

### 2.4 DNA extraction and high-throughput sequencing analyses

For each sample, 0.20 g of fresh straw material was used for genomic DNA isolation with the FastDNA^®^ SPIN Kit (MP Biomedicals, Illkirch, France), in conjunction with mechanical disruption via the FastPrep-24 instrument (MP Biomedicals, Irvine, CA). We used primer pairs 515F/806R (515F: 5’-GTGCCAGCMGCCGCGGTAA-3’; 806R: 5’-GGACTACVSGGGTATCTAAT-3’) and ITS3/ITS4 (ITS3: 5’-GCATCGATGAAGAACGCAGC-3’; ITS4: 5’-TCCTCCGCTTATTGATAT GC-3’) to amplify the bacterial V4 region and fungal ITS2 region, respectively. Amplification was carried out in triplicate for each sample using TransStart FastPfu DNA Polymerase to ensure technical reproducibility. Following PCR amplification, amplicons were cleaned and analyzed via paired-end sequencing. Raw sequence reads were processed using FLASH to assemble overlapping paired-end reads and Trimmomatic to remove low-quality bases and adapter sequences. Read pairs were merged only when they shared an overlap of at least 12 nucleotides with 100% sequence identity in the overlapping region. Putative chimeric sequences were identified and removed prior to downstream analysis. Bacterial amplicon sequence variants (ASVs) were classified taxonomically by referencing the SILVA database, while fungal ASVs were classified against the UNITE database. The complete set of raw sequencing reads has been archived in the NCBI Sequence Read Archive (SRA) and is accessible under the following accession numbers: PRJNA1327074 for bacterial communities and PRJNA1327092 for the fungal communities.

### 2.5 Statistical analyses

The rate of straw breakdown was determined as the proportional loss of litter mass relative to the original oven-dried wheat straw weight. Parallel assessments were conducted for the degradation dynamics of cellulose, hemicellulose, and lignin. Differences in decomposition rates and associated straw properties across treatments were evaluated using ANOVA.

Statistical computations were carried out via the Majorbio Cloud platform (https://www.majorbio.com). The α-diversity indices, including Shannon and Chao1, were computed to characterize microbial functional diversity.

To visualize and compare microbial community structures, principal coordinate analysis (PCoA) was conducted using Bray-Curtis dissimilarity indices for both bacterial and fungal taxa. To identify microbial taxa most responsive to decomposition progression, we regressed the relative abundances at genus-level for both bacteria and fungi against field decomposition time using the random forest approach (Bao et al., 2020). The importance of variables was assessed by measuring the decrease in model prediction accuracy following random permutation of each predictor.

Hierarchical partitioning and variation partitioning analyses were employed to quantify the individual and joint influences of environmental drivers on microbial community variation. First, the environmental variables were grouped into two categories: straw properties and community variation. Within each group, individual factors were ranked according to their explanatory power over microbial community structure. Subsequently, a stepwise selection procedure was implemented using the *ordistep* function in the *vegan* package in R, which identified the most parsimonious set of predictors from the full suite of environmental variables. The individual effect of each retained soil-related variable on the structure of microbial assemblages was subsequently quantified (Lai et al., 2022). Finally, partial least squares path modeling (PLS-PM) was used to evaluate the structural relationships among N fertilization treatments, sampling time, microbial community composition, enzyme activities, and straw decomposition dynamics.

## 3 Results

### 3.1 Wheat straw decomposition

**Figure 1:**
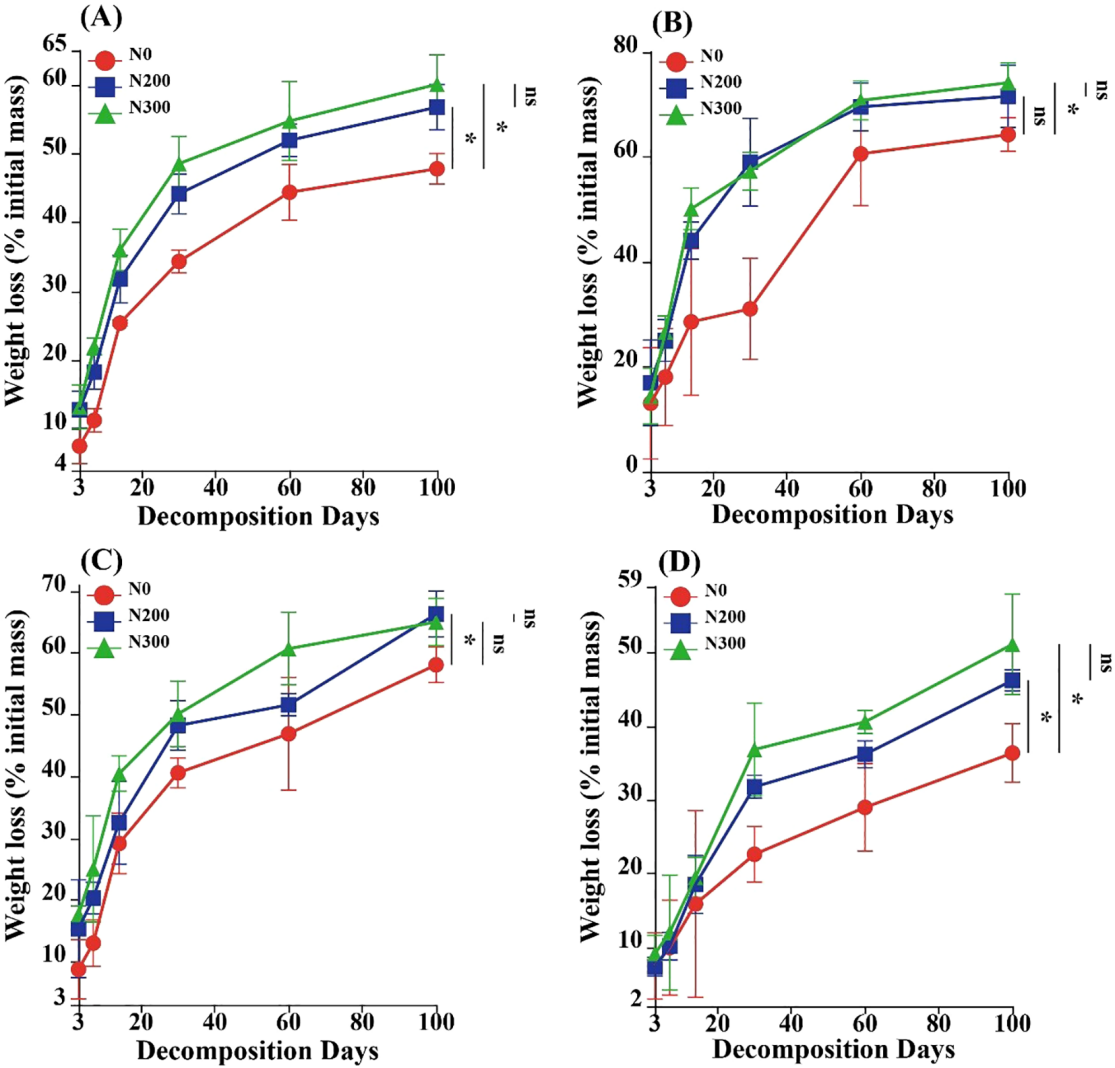
Mass loss dynamics of wheat straw **(A)**, cellulose **(B)**, hemicellulose **(C)**, and lignin **(D)** under three nitrogen fertilization treatments over 100 days of decomposition. Data represent means ± standard deviation (n=3). Asterisks (^*^) indicate significant differences between N treatments at *P* < 0.05 (Tukey’ s HSD test), and ns represents no significant difference.

Wheat straw mass loss, along with the degradation of cellulose, hemicellulose, and lignin, followed a consistent temporal pattern: an initial phase of rapid decomposition within the first 30 days, followed by a slower, more gradual decline thereafter, with the exception of cellulose under the N0 treatment, which exhibited a different trajectory (**Figure 1**). Nitrogen fertilization clearly influenced the overall decomposition process.

Relative to the N0 treatment, N addition (N200 and N300) significantly enhanced total straw mass loss (*P* < 0.05; **Figure 1A**). Specifically, the N200 treatment led to significantly greater hemicellulose degradation (*P* < 0.05; **Figure 1C**), while the N300 treatment significantly accelerated the breakdown of both cellulose and lignin (*P* < 0.05; **Figure 1B, D**). Nevertheless, no distinct differences were observed between N200 and N300 treatments in terms of mass loss for straw, cellulose, hemicellulose, or lignin. By the end of the incubation period, cumulative mass losses reached 47.84%, 56.80%, and 60.12% under N0, N200, and N300 treatments, respectively.

**Figure 2:**
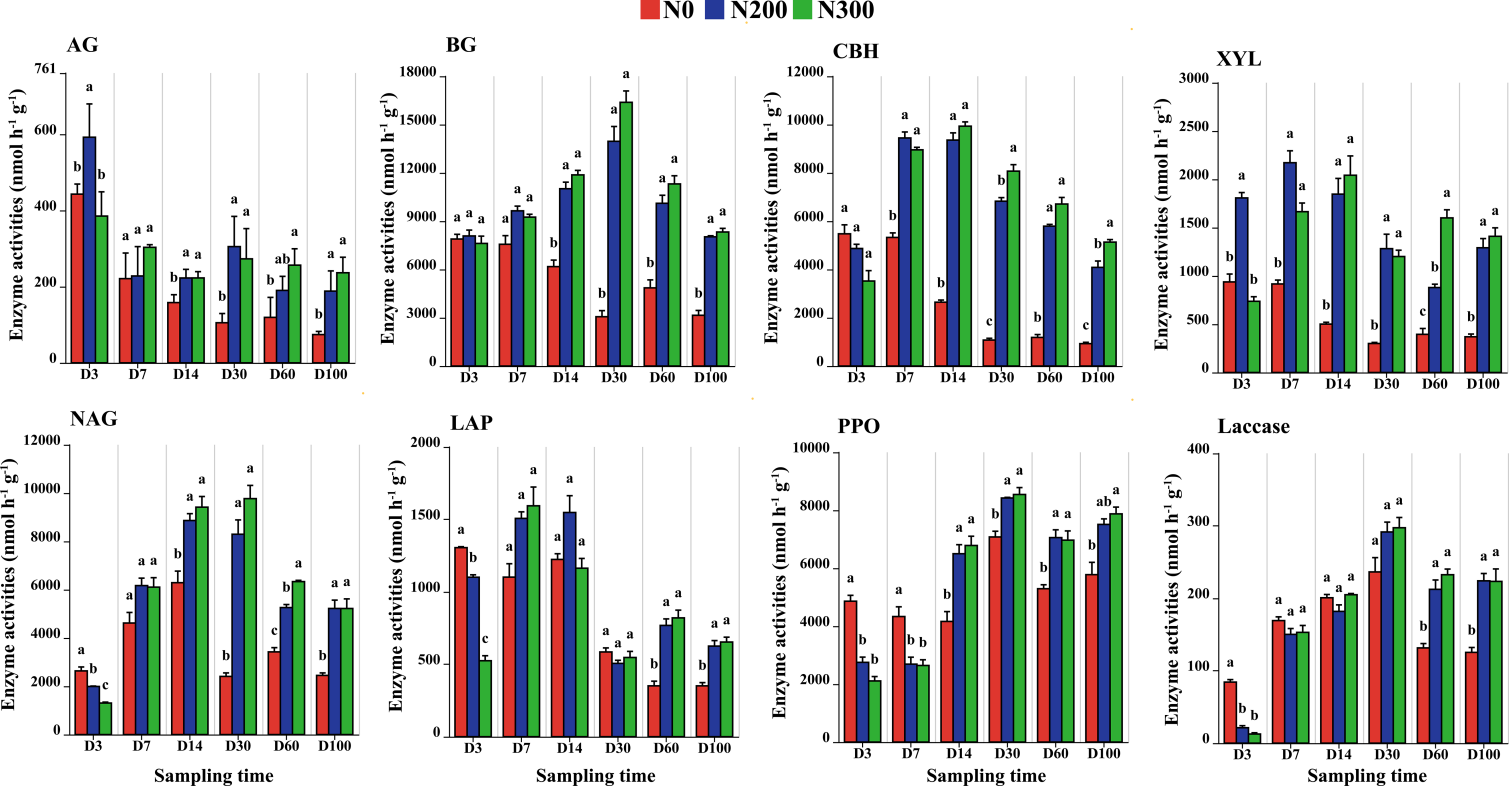
Temporal dynamics of extracellular enzyme activities (α-glucosidase: AG, β-glucosidase: BG, cellobiohyrolase: CBH, β-xylosidase: XYL, β-N-acetyl-glucosaminidase: NAG, leucine aminopeptidase: LAP and polyphenol oxidase: PPO) during straw decomposition. Values are means ± standard deviation (n=3). Different lower-case letters suggest significant differences among treatments at each sampling time (Tukey’ s HSD test, *P* < 0.05).

The enzyme activities associated with straw decomposition showed distinct temporal dynamics and responded differentially to nitrogen inputs. The activities of C-acquiring enzymes, α-glucosidase (AG) and β-glucosidase (BG), were consistently higher under N200 and N300 than under N0 from Day 14 to Day 100. Similarly, cellobiohyrolase (CBH) and β-xylosidase (XYL) activities were significantly enhanced under N-amended treatments from Day 7 to Day 100 (*P* < 0.05; **Figure 2**). For N-acquiring enzymes, β-N-acetylglucosaminidase (NAG) and leucine aminopeptidase (LAP) peaked under N0 at Day 3, but their activities subsequently became higher under N200 and N300. Specifically, NAG activity under N-amended treatments increased significantly from Day 14 onward, while LAP activity showed a significant rise only after Day 60 (*P* < 0.05; **Figure 2**). In contrast, oxidative enzyme activities were highest under N0 during the early decomposition phase: polyphenol oxidase (PPO) activity peaked at Days 3 and 7, and laccase activity was highest at Day 3 (*P* < 0.05). Compared with N0, N addition significantly increased PPO activity from Day 14 to Day 100 and elevated laccase activity at Days 60 and 100.

### 3.2 Succession of microbial communities during straw decomposition

As decomposition progressed, bacterial communities exhibited a steady increase in both Chao and Shannon indices, whereas fungal communities showed no consistent temporal trend (**Table 1**). Under the N0 treatment, the bacterial Chao index was significantly higher than under N200 and N300 at Days 3, 7, and 14 (*P* < 0.05). In contrast, the Shannon index for bacteria did not differ significantly among treatments at most sampling points, except at Day 3. For fungi, N addition (N200 and N300) significantly increased the Shannon index from Day 3 to Day 30 and increased the Chao index only at Day 3 compared with N0 (*P* < 0.05; **Table 1**).

**Table 1:** Temporal changes in bacterial and fungal α-diversity indices (Chao1 and Shannon) under N0 (no nitrogen), N200 (200 kg N ha^-1^) and N300 (300 kg N ha^-1^) treatments during straw decomposition.

Treatment		Bacterial diversity		Fungal diversity	
		Chao	Shannon	Chao	Shannon
Day3	N0	377.90 ± 62.03 a D	3.64 ± 0.38 a C	69.81 ± 4.88 b A	0.93 ± 0.01 c B
	N200	178.44 ± 74.35 b E	2.38 ± 0.53 b D	250.55 ± 34.65 a A	3.09 ± 0.22 a A
	N300	178.06 ± 22.57 b E	2.69 ± 0.40 b D	190.22 ± 40.16 a A	2.51 ± 0.16 b A
Day7	N0	422.17 ± 23.06 a CD	4.04 ± 0.30 a C	106.44 ± 8.38 a A	1.30 ± 0.17 b AB
	N200	316.94 ± 19.18 b DE	4.13 ± 0.10 a C	114.56 ± 7.63 a C	2.90 ± 0.31 a A
	N300	224.30 ± 61.18 b E	3.29 ± 0.70 a D	73.91 ± 35.94 a B	2.79 ± 0.36 a A
Day14	N0	710.31 ± 82.18 a C	4.88 ± 0.14 a B	70.76 ± 27.87 a A	1.53 ± 0.61 b AB
	N200	513.49 ± 47.42 b D	4.43 ± 0.35 a C	87.05 ± 4.52 a C	2.58 ± 0.46 a A
	N300	544.23 ± 21.63 b D	4.42 ± 0.00 a C	99.83 ± 27.88 a AB	2.33 ± 0.11 a A
Day30	N0	1366.80 ± 104.35 a B	5.70 ± 0.14 a A	96.99 ± 19.19 a A	1.84 ± 0.23 b AB
	N200	1249.12 ± 80.26 ab C	5.52 ± 0.03 a B	138.10 ± 8.49 a C	2.91 ± 0.16 a A
	N300	1054.58 ± 68.40 b C	5.15 ± 0.09 a BC	130.88 ± 30.78 a AB	2.70 ± 0.19 a A
Day60	N0	1737.08 ± 177.76 a A	6.05 ± 0.31 a A	150.33 ± 31.24 a A	2.36 ± 0.39 a A
	N200	1856.38 ± 185.04 a A	6.27 ± 0.14 a A	154.16 ± 51.44 a BC	2.80 ± 0.38 a A
	N300	1846.43 ± 87.96 a A	6.23 ± 0.14 a A	148.00± 35.71 a AB	2.67 ± 0.21 a A
Day100	N0	1651.43 ± 148.07 a AB	5.97 ± 0.26 a A	144.42 ± 65.52 a A	2.25 ± 0.78 a A
	N200	1560.28 ± 108.82 a B	6.16 ± 0.09 a AB	210.19 ± 9.28 a AB	3.22 ± 0.19 a A
	N300	1432.88 ± 110.08 a B	6.05 ± 0.19 a AB	159.68 ± 41.69 a AB	2.90 ± 0.22 a A

Data are mean ± standard deviation (SD) (n = 3). Different lower-case letters indicate significant differences among treatments at the same sampling time; upper-case letters suggest significant differences between across sampling times within the same treatment (Tukey’ s HSD test, *P* < 0.05).

At the phylum level, *Proteobacteria* and *Firmicutes* were the most abundant bacterial taxa, followed by *Actinobacteriota* and *Bacteroidota*. Together, these four phyla accounted for 68.80%-98.14% of all bacterial sequences across samples. In the N0 treatment, *Proteobacteria* dominated during the early decomposition stage (63.14% at Day 3, 63.76% at Day 7, and 63.35% at Day 14), but its relative abundance decreased as decomposition advanced (33.85%-46.34%). Under N200 and N300 treatments, *Proteobacteria* started at lower abundances but gradually became the primary phylum over time. *Firmicutes* emerged as the dominant bacterial group in nitrogen-amended treatments during the initial stages: it constituted 80.48% (N200) and 88.45% (N300) of sequences at Day 3, and 42.60% (N200) and 62.63% (N300) at Day 7. However, its abundance declined steadily as decomposition proceeded. In contrast, *Actinobacteriota* and *Bacteroidota* remained relatively stable throughout the experiment, with abundances ranging from 4.09% to 18.81% and 0.29% to 13.53%, respectively, across all samples.

Among fungi, *Ascomycota* and *Mucoromycota* consistently dominated across all treatments and sampling times, collectively representing 75.51%-98.98% of fungal sequences. *Basidiomycota* was mainly detected during the early (day 3) and late (Days 60 and 100) stages of decomposition.

**Figure 3:**
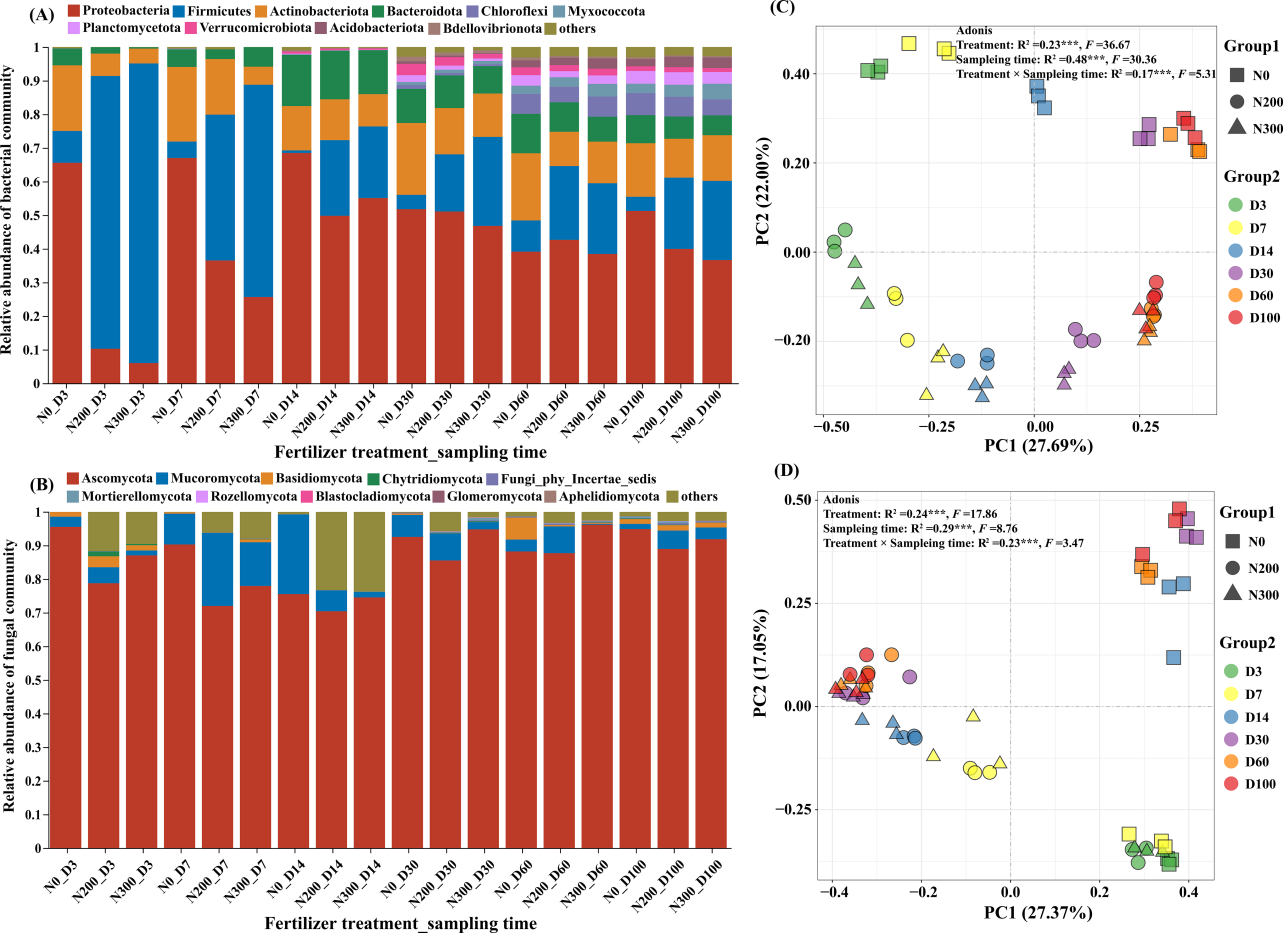
Relative abundance of bacterial **(A)** and fungal **(B)** communities at the phylum level across decomposition stages, and principle coordination analysis (PCoA) of bacterial **(C)** and fungal **(D)** community structure based on Bray-Curtis dissimilarity at the OTU level. Adonis analysis was applied using package “vegan” from R, with permutation = 999. *** represents P < 0.001.

Principal coordinate analysis (PCoA) based on Bray-Curtis dissimilarity revealed distinct clustering of microbial communities by both nitrogen treatment and sampling time (**Figure 3**). The first two axes explained 49.69% and 44.42% of the total variation in bacterial and fungal community composition, respectively. PERMANOVA (Adonis) further confirmed that both N fertilization and decomposition time significantly influenced microbial community structure (**Figure 3**; *P*<0.001).

### 3.3 Potential key microbial taxa for straw decomposition

**Figure 4:**
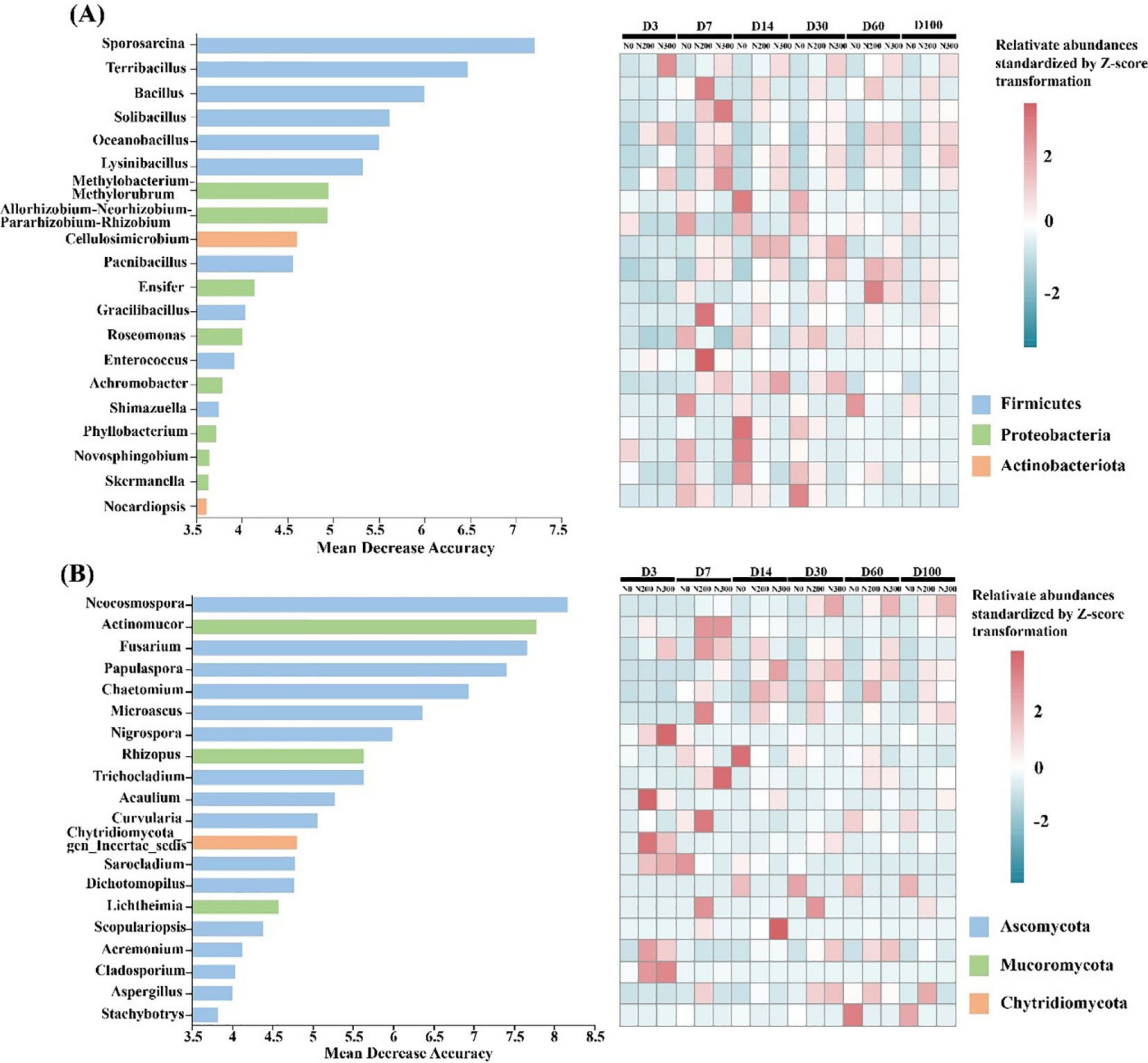
Top 20 bacterial **(A)** and fungal **(B)** genera identified as biomarkers of decomposition stage using random forest regression. Heatmaps show standardized relative abundances (Z-score transformed) across sampling time points under different N treatments. Z-score = (x-μ)/σ, where x, μ and σ denote for individual, average and standard deviation of relative abundance for each biomarker.

To identify microbial genera most strongly associated with decomposition progression under field conditions, we applied random forest regression to model the relationship between genus-level relative abundances and decomposition time. Model performance and variable importance were assessed using 10-fold cross-validation. The top 20 bacterial and fungal genera, ranked by time-discriminatory importance, were selected as biomarker taxa and are presented in **Figure 4**.

Among bacterial biomarkers, the majority belonged to the dominant phyla *Firmicutes*, *Proteobacteria*, and *Actinobacteria*. Notably, genera that exhibited higher relative abundances under N-amended treatments (N200 and N300) across decomposition stages, including *Prosarcina*, *Terribacillus*, *Bacillus*, and *Solibacillus*, were all classified within the class *Bacilli*. Two exceptions were *Cellulosimicrobium* (phylum *Actinobacteriota*) and *Achromobacter* (class *Gammaproteobacteria*), which also ranked among the top biomarkers under N addition. In contrast, biomarkers taxa enriched under the N0 treatment throughout decomposition were predominantly affiliated with *Alphaproteobacteria*. These included *Methylobacterim-Methylorubrum*, *Allorhizobium-Neorhizobium-Pararhizobium-Rhizobium*, *Roseomonas*, *Phyllobacterium*, *Novosphingobium*, *Skermanella*, and *Nocardiopsis*.

Among fungal biomarkers, 16 genera that showed higher relative abundances under N addition were distributed across multiple taxonomic classes: *Sordariomycetes* (*Neocosmospora*, *Fusarium*, *Papulaspora*, *Chaetomium*, *Microascus*, *Nigrospora*, *Trichocladium*, *Acaulium*, *Scopulariopsis*, and *Acremonium*), *Mucoromycetes* (*Actinomucor* and *Lichtheimia*), *Dothideomycetes* (*Curvularia* and *Cladosporium*), *Chytridiomycota_cls_Incertae_sedis* (*Chytridiomycota_gen_Incertae_sedis*), and *Eurotiomycetes* (*Aspergillus*). The remaining 4 biomarker genera, which were more abundant under N0, included *Rhizopus* (class *Mucoromycetes*) and three members of *Sordariomycetes* (*Sarocladium*, *Dichotomopilus*, and *Dichotomopilus*).

### 3.4 The relationships among residue properties, microbial communities and decomposition rate

**Figure 5:**
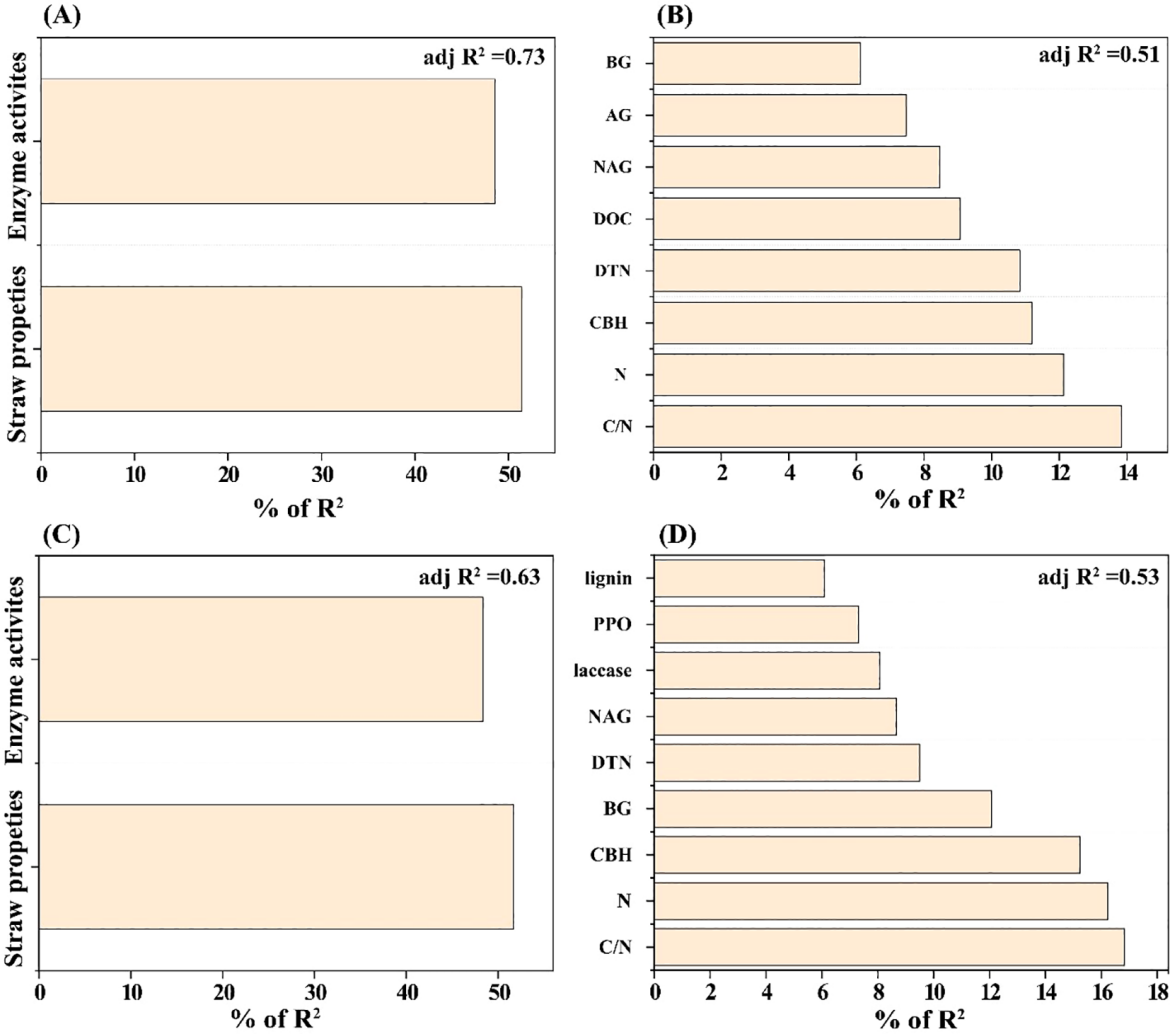
Hierarchical partitioning (HP) analysis quantifying the independent contributions of straw properties and enzyme activities to variation in bacterial **(A, B)** and fungal **(C, D)** community composition. **(A)** and **(C)** show variance explained by grouped factors; **(B)** and **(D)** show variance explained by individual factors. Values represent adjusted R².

Straw physicochemical properties and enzyme activities together explained 73.33% of the total variation in bacterial community composition (**Figure 5A**), with straw properties accounting for 51.41% and enzyme activities contributing 48.59%. Among individual drivers, the C/N ratio exerted the strongest influence, explaining 13.84% of the variance (*P*<0.01), followed by total N (12.13%, *P*<0.01), CBH (11.19%, *P*<0.01), DTN (10.83%, *P*<0.01), DOC (9.07%, *P*<0.01), NAG (8.47%, *P*<0.05), AG (7.47%, *P*<0.05), and BG (6.12%, *P*<0.05) (**Figure 5B**). For fungal communities, straw properties and enzyme activities collectively accounted for 62.99% of observed variations (**Figure 5C**), with straw properties contributing 51.66% and enzyme activities 48.34%. The C/N ratio, total N, and CBH were again the most influential factors, explaining 16.82% (*P*<0.01), 16.24% (*P*<0.01), and 15.24% (*P*<0.01) of the total variance, respectively. Additional significant contributors included BG (12.07%, *P*<0.01), DTN (9.5%, *P*<0.05), NAG (8.67%, *P*<0.05), laccase (8.07%, *P*<0.05), PPO (7.31%, *P*<0.05), and lignin content (6.08%, *P*<0.05) (**Figure 5D**).

**Figure 6:**
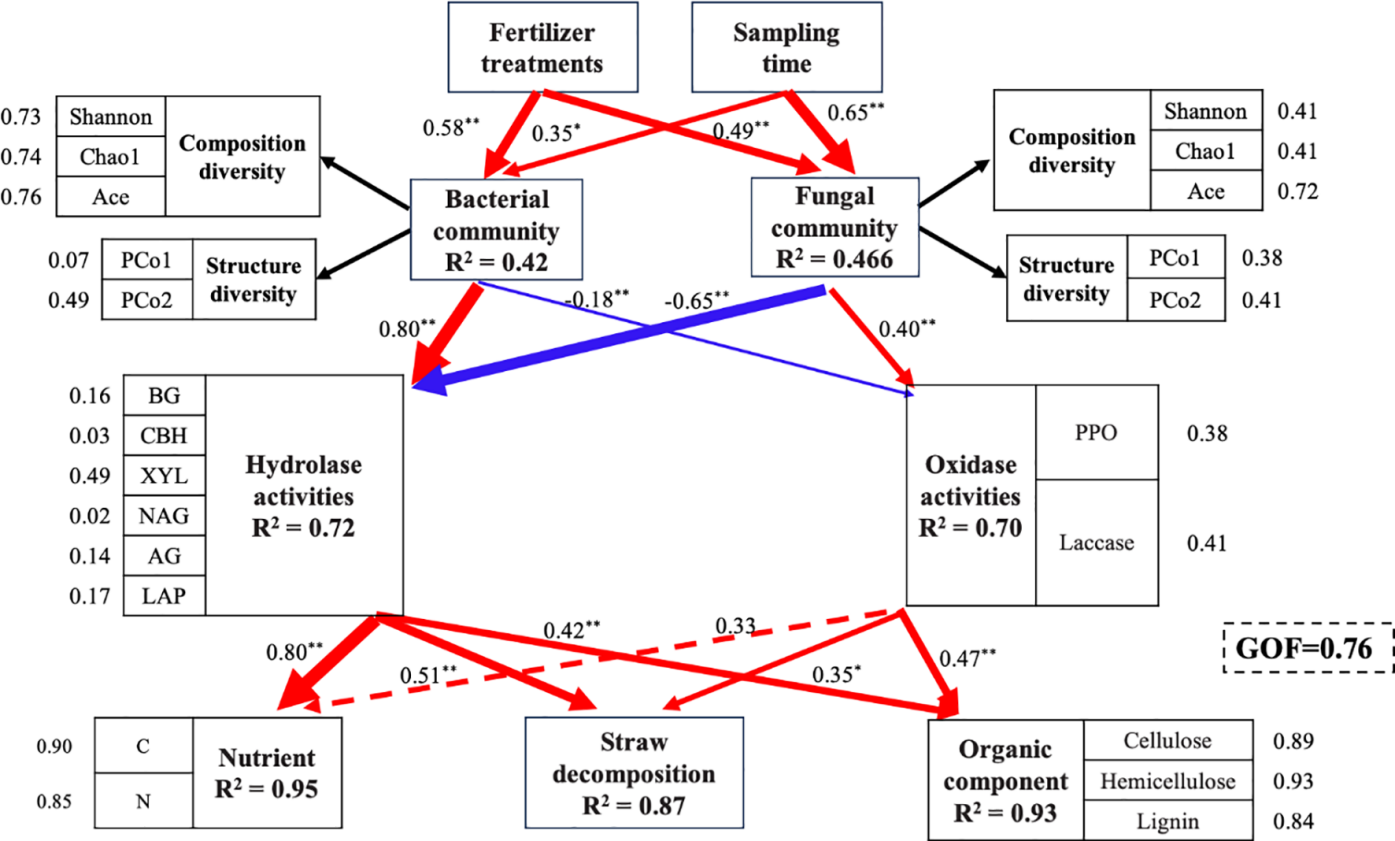
Partial least squares path model (PLS-PM) illustrating direct and indirect pathways linking N fertilization, sampling time, microbial diversity, enzyme activity, and straw decomposition rate. The model was evaluated based on the goodness of fit (GOF) index. Red and blue arrows represent significant positive and negative correlations, respectively, while the dotted gray arrows indicate no significant correlations. Numbers represent the standardized path coefficient, which are reflected by the thickness of arrows. Asterisks represent the level of significance, where * represents *P* < 0.05, ** represents *P* < 0.01.

To further explore the mechanistic pathways linking environmental factors, microbial communities, and decomposition dynamics, we conducted PLS-PM analysis (goodness-of-fit = 0.76; **Figure 6**). The results indicated that both N fertilization and sampling time exerted strong direct positive effects on the α- and β- diversity of bacterial and fungal communities. The bacteria diversity, in turn, significantly promoted hydrolase activity (standardized path coefficient = 0.80, *P*<0.01) while suppressing oxidase activity (-0.18, *P*<0.01). Fungal diversity showed the opposite pattern, positively influencing oxidase activity (0.40, *P*<0.01) and negatively affecting hydrolase activity (-0.65, *P*<0.01). Ultimately, both hydrolase (0.51, *P*<0.01) and oxidase (0.35, *P*<0.01) activities contributed positively to the overall rate of straw decomposition.

## 4 Discussion

### 4.1 Effect of N fertilization on wheat straw decomposition

This study centered on the dynamic decomposition phase of wheat straw residues, during which 47.84%- 60.12% of initial straw mass was lost across all treatments within 100 days. These findings align closely with prior reports; Gao et al. (2016) observed approximately 50% mass loss over 110 days, while Zhong et al. (2020) documented a 54.8% reduction over 4 months. The temporal pattern of mass loss in our experiment also mirrors trends reported in prior studies on crop straw and other plant litter types (Wu et al., 2009; Zhong et al., 2020).

Notably, both N200 and N300 treatments resulted in significantly greater mass loss compared with N0 (*P*<0.05), confirming that N addition exerts strong impacts on decomposition dynamics (Mühlbachová et al., 2021; Zhang et al., 2025). Meta-analyses have previously indicated that N inputs ranging from 7.5 to 12.5 g N m^-2^ yr^-1^ generally accelerate decomposition across ecosystems (Knorr et al., 2005; Zhang et al., 2018), a range that encompasses the application rates used in this study. It is widely recognized that N fertilization promotes residue decomposition in agricultural soils through providing sufficient N sources for decomposer communities. Soil enzymes serve as primary mediators of plant residue degradation in most terrestrial ecosystems (Koeck et al., 2014). Previous studies have shown that N deficiency can inhibit enzyme synthesis, thereby limiting microbial capacity to utilize available C and exacerbating C limitation (Sinsabaugh et al., 2008). In contrast to this expectation, we observed that N-acquiring enzymes activities were highest under N0 at the earliest sampling point (Day 3; *P*<0.05). This may reflect a microbial strategy to reallocate carbon resources toward the production of N-scavenging enzymes when inorganic N is scarce, thereby enhancing access to organic N pools (Marklein and Houlton, 2012). Overall, N addition significantly elevated the activities of key hydrolytic and oxidative enzymes during the majority of the decomposition period. This enzymatic stimulation likely underpinned the accelerated mass loss observed under N200 and N300, as enzyme activities are significantly linked to litter decay rates and changes in litter biochemical quality (Song et al., 2016). However, the optimal nitrogen application rate should be determined through a comprehensive analysis of straw decomposition and crop yield in further research.

### 4.2 Microbial community response to N status during residue decomposition

Crop residue decomposition is the primary pathway for organic matter input in agricultural ecosystems and is fundamentally mediated by microbial activity (Gao et al., 2016). Our findings offer important insights into the interplay between straw degradation dynamics and the successional trajectories of associated microbial communities. As expected, both bacterial and fungal assemblages colonizing wheat straw exhibited distinct temporal shifts, with community structure significantly affected by N fertilization and sampling time, as confirmed by PCoA analysis. This successional pattern reflects two interrelated drivers. First, biochemical decomposition follows a sequential trajectory: early stages are dominated by the breakdown of labile components (e.g., water-soluble constituents, hemicellulose, and unprotected cellulose), followed by the slower degradation of recalcitrant polymers (e.g., lignin or suberin) (Klotzbucher et al., 2011). These substrate transitions are mirrored by corresponding changes in the composition and function of litter-associated microbial communities (Dilly et al., 2001). Second, N fertilization directly modulated the abundance of specific microbial taxa throughout decomposition. Higher N availability, coupled with a reduced lignin-to-nitrogen ratio, likely facilitated more rapid microbial colonization of straw under N-amended treatments compared with N0.

In our study, *Firmicutes* and *Proteobacteria* emerged as the most abundant bacterial phyla, collectively accounting for over 50% of all sequences and widely recognized for their key roles in organic matter degradation (Jurado et al., 2014; Li et al., 2023). *Bacteroidota* and *Actinobacteriota*, which ranked next in relative abundance, are also frequently associated with lignocellulose decomposition (Wang et al., 2024; Xiang et al., 2024). Notably, *Firmicutes* dominated the bacterial community during the initial decomposition phase (Days 3 and 7), particularly under N addition. This pattern suggests that microbial community is strongly governed by resource availability and nutritional stoichiometry (Zheng et al., 2021). Prior studies indicate that *Firmicutes*, often characterized as fast-growing copiotrophs employing *r*-selected strategies, are preferentially enriched in environments rich in labile C (Fierer et al., 2007). As the DOC concentration declined and recalcitrant substrates accumulated, their dominance of gradually gave way to taxa better adapted to oligotrophic or lignin-rich conditions. Among fungi, *Ascomycota* was the predominant phylum throughout decomposition, surpassing *Mucoromycota* and *Basidiomycota* in relative abundance. All three phyla are well-documented as key agents of straw decomposition, possessing enzymatic machinery capable of degrading lignocellulosic and lignin-rich substrates (Wang et al., 2025).

Due to the strong temporal coupling between decomposition progression and microbial community dynamics, we employed random forest modeling to identify 20 bacterial and fungal genera that serve as biomarkers of community succession during straw decomposition. Among bacterial biomarkers, genera such as *Terribacillus*, *Bacillus*, *Solibacillus*, *Oceanobacillus*, and *Cellulosimicrobium* are well-documented lignocellulose degraders. Previous studies have confirmed their ability to secrete extracellular hydrolases that catalyze the depolymerization of complex plant polymers and accelerate organic matter turnover (López et al., 2021; Lewin et al., 2022; Du et al., 2025; Liu et al., 2025). Similarly, key fungal biomarkers, such as *Neocosmospora*, *Actinomucor*, *Fusarium*, *Chaetomium*, *Aspergillus*, and *Cladosporium* are known for their strong enzymatic machinery, particularly their production of xylanases, laccases, and ligninolytic peroxidases, which target recalcitrant aromatic and polymeric substrates (Moreno et al., 2021; Tõlgo et al., 2021; Ibrahim et al., 2022; Wang et al., 2023). The enrichment of these taxa under nitrogen-amended treatments likely contributes to the observed increases in enzyme activities and accelerated decomposition rates. In addition to decomposers, several N-fixing bacterial genera, including *Allorhizobium-Neorhizobium-Pararhizobium-Rhizobium*, *Novosphingobium*, *Skermanella*, and *Enifer*, were also identified as biomarkers, particularly under low-nitrogen conditions. These taxa are known to assimilate atmospheric nitrogen during decomposition, thereby alleviating nitrogen limitation and facilitating the activity of secondary decomposers (Tláskal et al., 2021). Under nitrogen-deficient conditions, such as in the N0 treatment, these diazotrophs may serve as critical nitrogen donors to fungal communities, supporting their metabolic activity and enzymatic output (Zhang et al., 2022). This functional complementarity suggests that N-fixing bacteria plays a supportive, yet essential, role in sustaining decomposition processes when external nitrogen inputs are limited.

In this study, we evaluated the independent explanatory power of multiple environmental factors to identify key drivers shaping microbial community structure during straw decomposition. Our results indicate that N fertilization significantly altered straw properties, particularly the C/N ratio and TN content, which emerged as the most influential regulators of both bacterial and fungal community composition. This finding is consistent with recent meta-analytical evidence suggesting that the C/N ratio serves as a primary control on residue decomposition dynamics across diverse biomes (Miao et al., 2025; Guo et al., 2022). Nitrogen, as an essential nutrient for microbial metabolism, directly supports the biosynthesis of essential cellular components, including proteins, enzymes, nucleotides, and secondary metabolites, all of which are critical for microbial growth and function (He et al., 2023). Chen et al. (2024) further showed that higher soil NO_3_^–^N levels correlate with a significant decline in residue C/N during decomposition, which in turn drives shifts in bacterial community structure and module I composition. In addition to bulk stoichiometry, both labile and recalcitrant carbon pools exhibited strong associations with microbial community structure. DOC and DTN were closely linked to bacterial assemblages, whereas lignin content showed stronger correlations with fungal communities. This pattern likely reflects the distinct substrate preferences of bacteria and fungi; bacteria typically thrive on soluble, readily available substrates, while fungi are better adapted to degrade complex, recalcitrant polymers (Meidute et al., 2008; Zhou et al., 2017). This functional differentiation is further confirmed by our finding that the PPO and laccases were significantly correlated with fungal community composition. Notably, CBH displayed the strongest correlation with overall microbial community structure. As a rate-limiting enzyme in cellulose hydrolysis, CBH plays a key role in regulating C flux during decomposition by initiating the breakdown of crystalline cellulose into bioavailable sugars (Chen et al., 2023). Integration of these findings with the PLS-PM analysis, it reveals a coherent mechanistic pathway: N fertilization reduces residue C/N, which in turn reshapes bacterial and fungal community composition and enhances the production of key hydrolytic and oxidative enzymes, thereby accelerating the residue decomposition process (Nannipieri et al., 2012; Chen et al., 2024).

Collectively, our findings illuminate the microbial mechanisms driving wheat straw decomposition under contrasting N regimes. However, this study did not assess the expression of functional genes directly involved in lignocellulose degradation. Future investigations employing metagenomic approaches would provide deeper mechanistic insights into the genetic potential and *in situ* activity of microbial communities.

## 5 Conclusion

In this study, we investigated the straw decomposition rate, C component decomposition, enzyme activities and the microbial community that associated with straw decomposition under different rate N fertilizer application. Strong evidence as the improvement of mass loss and cellulose, hemicellulose and lignin decomposition proved that N fertilization could accelerate wheat straw decomposition. Bacterial taxa *Proteobacteria*, *Firmicutes*, *Actinobacteriota* and *Bacteroidota*, and fungal taxa *Ascomycota*, *Mucoromycota* and *Basidiomycota* were the predominant members for straw decomposition. Moreover, distinct successional pattern of straw decomposing bacterial and fungal community composition was observed under different N input, which was tightly coupled with shifts in straw physicochemical properties, especially the C/N ratio, TN content, and CBH activity. Importantly, biomarker taxa including bacterial genera *Bacillus* and *Cellulosimicrobium*, and fungal genera *Fusarium*, *Chaetomium*, and *Aspergillus* were specifically identified as responsive for enhanced decomposition efficiency under nitrogen-amended conditions. Therefore, we assumed that N fertilization reduced residue C/N, which in turn triggered compositional shifts in microbial communities and stimulated the production of hydrolytic and oxidative enzymes, ultimately accelerating the decomposition of wheat straw. In the future, these potentially functional microbial strains for straw decomposition are necessary to be isolated and used for *in situ* straw decomposition promotion.

## Data Availability

The datasets presented in this study can be found in online repositories. The names of the repository/repositories and accession number(s) can be found below: https://www.ncbi.nlm.nih.gov/, PRJNA1327074 https://www.ncbi.nlm.nih.gov/, xPRJNA1327092.
