# Supplementary material for: Nitrogen addition accelerated straw in-situ decomposition by promoting specific microbial taxa growth and straw decomposing enzyme activities
Source: Front Plant Sci. 2025 Dec 17;16:1703916. doi: 10.3389/fpls.2025.1703916 (PMC12753900; doi:10.3389/fpls.2025.1703916)
Supplement: Supplementary file 1 [file Table1.docx]

**Supplementary file**

**Title:** Nitrogen addition accelerated straw in-situ decomposition by promoting specific microbial taxa growth and straw decomposing enzyme activities

**Authors:** Tengfei Guo^a^, Mengyuan Wang^a^, Yulu Chen^a^, Ke Yue^a^, Long Ma^b^, Shaomin Huang^a*^, Xinpeng Xu^b*^, Xiao Song^a^, Sumiao Su^a^, Zekun Zhang^a^, Qian Zhang^c^, Keke Zhang^d^

^a^ *Institution of Plant Nutrition and Environmental Resources,* *Henan Academy of Agricultural Sciences, Zhengzhou, 450002, PR, China.*

^b^ *State Key Laboratory of Efficient Utilization of Arid and Semi-arid Arable Land in Northern China/Key Laboratory of Plant Nutrition and Fertilizer, Ministry of Agriculture and Rural Affairs,* *Institute of Agricultural Resources and Regional Planning, Chinese Academy of Agricultural Sciences, Beijing 100081, PR, China.*

^c^ *Resources and Environment College, Henan Agricultural University, Zhengzhou, 450002, PR China*.

^d^ *Institution of Edible Fungi, Henan Academy of Agricultural Sciences, Zhengzhou, 450002, PR, China.*

* Corresponding author: Institution of Plant Nutrition and Environmental Resources,

Henan Academy of Agricultural Sciences, Zhengzhou, 450002, PR, China.

Post address: No. 116, Huayuan Road, Zhengzhou, Henan Province.

E-mail address: hsm503@126.cn

* Corresponding author: Institute of Agricultural Resources and Regional Planning,

Chinese Academy of Agricultural Sciences, Beijing 100081, PR, China.

Post address: No. 12, Zhongguancun South Street, Beijing, China.

E-mail address: xuxinpeng@caas.cn

**Table S1.** Straw residue properties along the decomposition time under different fertilization treatments. Data represent means ± S.D.; n = 3. Different lowercase in same column indicates significant differences among different fertilization at the same sampling time (Turkey’s HSD test, *P* < 0.05).

|  |  | TC  (%) | TN  (%) | C/N | DOC  (g kg^-1^) | DTN  (g kg^-1^) |
| --- | --- | --- | --- | --- | --- | --- |
| Day 3 | N0 | 42.36 ± 0.27 a | 0.49 ± 0.04 b | 87.53 ± 6.65 a | 8.16 ± 1.21 b | 0.33 ± 0.07 b |
|  | N200 | 42.13 ± 0.11 a | 0.64 ± 0.05 a | 66.04 ± 4.84 b | 13.80 ± 3.98 ab | 1.49 ± 0.45 a |
|  | N300 | 42.40 ± 0.21 a | 0.67 ± 0.04 a | 63.80 ± 3.94 b | 17.03 ± 1.28 a | 1.95 ± 0.10 a |
|  |  |  |  |  |  |  |
| Day 7 | N0 | 42.29 ± 0.12 a | 0.56 ± 0.05 b | 75.57 ± 7.86 a | 14.58 ± 2.17 a | 0.69 ± 0.21 b |
|  | N200 | 42.22 ± 0.21 a | 1.03 ± 0.02 a | 40.82 ± 0.93 b | 20.90 ± 3.79 a | 2.09 ± 0.30 a |
|  | N300 | 41.85 ± 0.05 a | 1.08 ± 0.07 a | 38.69 ± 2.49 b | 16.82 ± 1.99 a | 2.08 ± 0.07 a |
|  |  |  |  |  |  |  |
| Day 14 | N0 | 42.31 ± 0.11 b | 0.60 ± 0.02 b | 70.71 ± 2.83 a | 11.18 ± 1.64 a | 0.61 ± 0.07 a |
|  | N200 | 43.01 ± 0.22 a | 1.16 ± 0.10 a | 37.20 ± 3.16 b | 11.45 ± 2.58 a | 1.06 ± 0.33 a |
|  | N300 | 42.89 ± 0.29 a | 1.20 ± 0.08 a | 35.75 ± 2.09 b | 11.38 ± 3.89 a | 1.12 ± 0.24 a |
|  |  |  |  |  |  |  |
| Day 30 | N0 | 43.01 ± 0.30 a | 0.63 ± 0.06 b | 68.26 ± 7.33 a | 3.38 ± 0.97 b | 0.20 ± 0.08 b |
|  | N200 | 42.81 ± 0.68 a | 1.33 ± 0.10 a | 32.39 ± 2.79 b | 6.98 ± 1.14 a | 0.62 ± 0.04 a |
|  | N300 | 43.11 ± 0.17 a | 1.41 ± 0.12 a | 30.81 ± 2.48 b | 7.24 ± 0.89 a | 0.69 ± 0.17 a |
|  |  |  |  |  |  |  |
| Day 60 | N0 | 42.92 ± 0.37 a | 0.81 ± 0.06 b | 53.09 ± 3.64 a | 4.27 ± 0.46 b | 0.33 ± 0.13 a |
|  | N200 | 42.74 ± 0.87 a | 1.56 ± 0.10 a | 27.41 ± 2.05 b | 5.35 ± 0.78 ab | 0.48 ± 0.06 a |
|  | N300 | 41.80 ± 1.25 a | 1.82 ± 0.34 a | 23.54 ± 4.58 b | 6.10 ± 0.75 a | 0.72 ± 0.26 a |
|  |  |  |  |  |  |  |
| Day 100 | N0 | 41.66 ± 1.20 a | 0.95 ± 0.04 b | 43.97 ± 2.55 a | 3.75 ± 0.19 a | 0.27 ± 0.03 b |
|  | N200 | 41.79 ± 0.02 a | 1.77 ± 0.08 a | 23.68 ± 1.12 b | 4.65 ± 0.74 a | 0.42 ± 0.09 ab |
|  | N300 | 42.44 ± 0.29 a | 1.90 ± 0.18 a | 22.50 ± 2.21 b | 6.47 ± 2.21 a | 0.62 ± 0.22 a |
